# Impact of multimodal AI literacy, critical thinking, and resilience on academic performance in early childhood education: a multiple chain mediation analysis of multimodal AI self-efficacy and cognitive engagement

**DOI:** 10.3389/fpsyg.2026.1873827

**Published:** 2026-07-02

**Authors:** Wu Lei, Kamariah Abu Bakar, Khairul Farhah Khairuddin, Juan Du

**Affiliations:** 1 Universiti Kebangsaan Malaysia, Bangi, Malaysia; 2 Monash University Malaysia, Bandar Sunway, Malaysia

**Keywords:** cognitive engagement, early childhood education, multimodal AI literacy, multimodal AI self-efficacy, resilience

## Abstract

**Introduction:**

With the continuous integration of multimodal artificial intelligence (AI) into higher education, students majoring in Early Childhood Education (ECE) are expected not only to master the operational use of multimodal AI tools but also to develop the ability to evaluate, reflect on, and adapt to intelligent learning environments. However, existing studies have mainly focused on engineering and information technology contexts, while limited attention has been paid to ECE students and to the mechanisms through which multimodal AI self-efficacy and cognitive engagement influence academic achievement.

**Methods:**

This study focused on university students majoring in ECE and collected 458 valid responses through a questionnaire survey. Partial Least Squares Structural Equation Modeling (PLS-SEM) was used to examine the effects of multimodal AI literacy, critical thinking, and resilience on academic achievement, as well as the mediating roles of multimodal AI self-efficacy and cognitive engagement.

**Results:**

The results showed that multimodal AI literacy, critical thinking, and resilience had significant positive effects on academic achievement. Multimodal AI self-efficacy and cognitive engagement played important mediating roles in the relationships between these antecedent variables and academic achievement. Among the antecedents, critical thinking had the strongest effects on both cognitive engagement and multimodal AI self-efficacy, whereas the direct effect of resilience on cognitive engagement was not significant.

**Discussion:**

These findings enrich the theoretical understanding of how academic achievement is formed in AI-supported educational contexts. The study also provides practical implications for universities seeking to optimize ECE curricula, enhance students’ multimodal AI application skills, and cultivate their broader competencies for learning and professional development in intelligent educational environments.

## 1 Introduction

In the context of digital transformation and the rapid development of intelligent technologies, artificial intelligence (AI) is profoundly reshaping the learning ecology and educational practices of higher education worldwide (Peng and Li, 2025). Existing research has indicated that AI not only changes the ways in which students acquire knowledge and process information but also promotes continuous innovation in instructional design, learning support, and assessment methods (Yuan and Li, 2025; Marcos, 2025). With the widespread adoption of multimodal AI and intelligent learning systems, university students’ learning is gradually shifting from passive reception of information to more personalized, interactive, and data-driven learning approaches. These technologies can provide customized learning experiences and enhance the accessibility of learning resources, thereby effectively improving learning efficiency and engagement (Adiguzel et al., 2023). However, as multimodal AI increasingly integrates into learning processes, students majoring in Early Childhood Education (ECE) face growing challenges. They not only need to master the basic operational skills of multimodal AI tools but also require the ability to apply these tools judiciously in professional learning and future teaching practice. For example, ECE students’ use of multimodal AI is mainly reflected in professional learning tasks, such as generating and evaluating texts, images, stories, videos, and teaching activity materials; however, they often find it difficult to determine whether AI-generated content aligns with children’s developmental characteristics (Holmes and Porayska-Pomsta, 2022). This ability is particularly critical for ECE students, as they are not only current learners but also future practitioners whose multimodal AI literacy and critical thinking will directly influence the pedagogical philosophies and learning environment constructions in early childhood education. Therefore, systematically exploring the impact of multimodal AI-related competencies on academic performance in ECE students within higher education contexts constitutes a research topic of significant theoretical and practical value.

When examining the key factors affecting academic performance in multimodal AI contexts, this study further focuses on three core competencies: multimodal AI literacy, critical thinking, and resilience. An analytical framework is constructed from three dimensions—technical literacy, cognitive judgment, and psychological adaptation—to investigate how these core competencies influence learning outcomes among ECE students. First, multimodal AI literacy is defined as learners’ comprehensive ability to understand, effectively use, and evaluate multimodal AI tools. Its importance lies in the fact that only with sufficient multimodal AI literacy can students transition from passively using technology to proactively optimizing learning strategies, thereby enhancing learning effectiveness (Ng et al., 2021). Second, critical thinking emphasizes learners’ ability to analyze and evaluate AI-generated content, which is crucial for discerning information reliability and avoiding blind trust or overreliance on algorithmic outputs, particularly in the context of the growing prevalence of multimodal AI (Zhai et al., 2024). Third, resilience reflects an individual’s capacity to adapt and regulate under technological complexity and learning pressure, supporting sustained engagement and overcoming setbacks in dynamic digital learning environments (Ge, 2025). Existing studies indicate that psychological factors, such as self-efficacy and learning motivation, significantly influence learning engagement and outcomes (Hanham et al., 2021), while research in the technology acceptance domain highlights the role of individual capabilities and cognitive expectations in shaping the use of new technologies (Bai and Yang, 2025).

Based on the above research background and identified gaps, this study aims to construct and test a comprehensive model to systematically investigate how multimodal AI literacy, critical thinking, and resilience influence academic performance among ECE students through multimodal AI self-efficacy and cognitive engagement. Compared with prior studies primarily focusing on technology adoption intentions, the theoretical contribution of this study lies in extending multimodal AI literacy to the ECE context, emphasizing the integrated competency structure required by future educators in the era of multimodal AI. Simultaneously, by introducing multimodal AI self-efficacy and cognitive engagement as mediating variables, this study enriches understanding of the psychological and cognitive mechanisms underlying learning processes. Practically, it also aims to provide empirical evidence for universities to cultivate critically thinking and adaptable ECE talents and to offer guidance for integrating AI into teacher education curriculum design.

The structure of this paper is as follows: Section 2 reviews relevant literature and presents the theoretical foundation and analytical framework of this study; Section 3 introduces the research design and methodology, including the sample, measurement instruments, and data analysis procedures; Section 4 reports the empirical findings and further discusses their theoretical and practical implications; finally, Section 5 summarizes the study’s main contributions and limitations and proposes directions for future research.

## 2 Literature review

With the gradual integration of multimodal AI into higher education, the factors influencing academic performance have become a critical topic in educational research. Existing studies generally indicate that learners’ technological skills, psychological factors, and learning engagement are key variables affecting academic performance (Zakir et al., 2025). For example, some research emphasizes that digital literacy and technology usage skills can improve students’ efficiency in acquiring information and solving problems, thereby promoting learning outcomes (Zakir et al., 2025). Other studies, from the perspective of technology acceptance, suggest that perceived usefulness, self-efficacy, and learning motivation influence students’ engagement in intelligent learning environments (Chen, 2025). In addition, cognitive engagement and deep learning strategies have been shown to be closely associated with academic performance, as active cognitive investment facilitates knowledge integration and higher-order thinking development. However, existing studies have mainly focused on engineering, information technology, or natural science disciplines, while insufficient attention has been paid to the learning characteristics of students majoring in Early Childhood Education and Child Development in AI-supported environments (Sanusi et al., 2024). Meanwhile, with the increasing prevalence of multimodal artificial intelligence, researchers have begun to emphasize the importance of critical thinking and learning adaptability, suggesting that students need the ability to evaluate the quality of AI-generated outputs and cope with learning pressure brought about by technological change. However, relevant empirical research remains limited (Asio, 2024). Overall, existing studies have primarily focused on AI usage intention or technology acceptance, with limited explanation of how AI literacy, critical thinking, and resilience influence academic performance. Therefore, this study takes multimodal AI literacy, critical thinking, and resilience as core independent variables, integrating multimodal AI self-efficacy and cognitive engagement to systematically explore their effects on ECE students’ academic performance.

### 2.1 Theoretical basis of this study

#### 2.1.1 Social cognitive theory

Social Cognitive Theory (SCT), proposed by Albert Bandura (1986), emphasizes the reciprocal interactions among individual factors, behavior, and the environment, suggesting that learning arises not only from external stimuli but also from cognitive beliefs and self-regulatory capacities (Stajkovic and Sergent, 2019). Previous research has shown that self-efficacy, as a core construct of SCT, is a key determinant of academic performance (Krcmar, 2019). Social Cognitive Theory provides the theoretical foundation for explaining the relationships among personal competencies, cognitive beliefs, learning behaviours, and learning outcomes among ECE students in multimodal AI-supported learning contexts. In this study, multimodal AI literacy, critical thinking, resilience, and multimodal AI self-efficacy are conceptualized as students’ personal factors. Specifically, multimodal AI literacy reflects students’ ability to understand, evaluate, and apply AI-generated multimodal resources; critical thinking represents students’ capacity to analyse and judge the reliability and pedagogical appropriateness of AI-generated content; resilience reflects students’ ability to adapt and sustain effort when facing technological uncertainty and learning challenges; and multimodal AI self-efficacy represents students’ belief in their capability to effectively use multimodal AI to complete academic and professional learning tasks. Cognitive engagement is regarded as the behavioural learning process because it reflects students’ deep mental investment, active processing, reflection, and sustained effort in learning. Academic performance is treated as the learning outcome, reflecting the extent to which students complete academic tasks and achieve desirable learning results. Meanwhile, the multimodal AI-supported ECE learning context constitutes the environmental condition of this study, in which students interact with AI-generated texts, images, videos, stories, and teaching materials. Therefore, Social Cognitive Theory is appropriate for this study because it explains how students’ competencies and beliefs are transformed into learning engagement and ultimately influence academic performance.

### 2.2 Multimodal AI literacy and academic performance

Multimodal AI literacy generally refers to an individual’s knowledge, skills, and judgment to understand, evaluate, and effectively use AI technologies (Xiao et al., 2024). In higher education contexts, some studies have begun to directly examine the effects of AI literacy on learning outcomes. Xiao et al. (2024), studying 400 undergraduate students, found that AI literacy had a direct effect on academic performance, indicating that higher AI literacy is associated with better learning outcomes. In this study, multimodal AI literacy is defined as students’ ability to understand, use, evaluate, and ethically apply multimodal AI resources, including text, images, audio, and video.

*H1*: Multimodal AI Literacy has a significant positive effect on Academic Performance.

### 2.3 Critical thinking and academic performance

Critical thinking refers to higher-order cognitive abilities that involve analyzing, evaluating, reasoning, and judging information (Facione et al., 1994). Previous studies have indicated a significant association between critical thinking and academic performance, critical thinking is important for evaluating AI-generated content, avoiding over-reliance on AI, and promoting deep learning; however, empirical examination of this construct among ECE students remains limited (Zhai et al., 2024). In this study, critical thinking is defined as students’ ability to analyse, evaluate, and judge the reliability and pedagogical appropriateness of AI-generated information. Ren et al. (2020) found that critical thinking could significantly predict academic performance even after controlling for general cognitive ability, indicating that critical thinking independently contributes to learning outcomes. Additionally, a meta-analysis by Orhan (2022) integrating 47 independent studies and 67 datasets demonstrated a moderate positive correlation between critical thinking and academic performance, further confirming a stable relationship between the two.

*H2*: Critical Thinking has a significant effect on Academic Performance.

### 2.4 Resilience and academic performance

Resilience refers to an individual’s ability to maintain adaptation, recover, and continue exerting effort when facing stress, setbacks, and challenges (Windle, 2011). Studies have shown a positive relationship between resilience and academic performance. Cai and Meng (2025), in a study of university students, reported a significant positive correlation between academic resilience and academic performance, supporting this hypothesis. In this study, resilience is defined as students’ ability to maintain adaptation and sustain effort when facing uncertainty, difficulties, and pressure in AI-supported learning.

*H3*: Resilience has a significant effect on Academic Performance.

### 2.5 Mediating role of multimodal AI self-efficacy

Asio (2024) found significant relationships among AI literacy, AI self-efficacy, and AI competence among university students. Students with higher AI literacy generally possess greater understanding of AI tools’ functions, limitations, risks, and applications, which enables them to correctly judge operational procedures, screen outputs, and resolve issues during AI tasks. According to self-efficacy theory, mastery of relevant knowledge and skills strengthens individuals’ beliefs in their task competence. Therefore, students with higher multimodal AI literacy are more likely to develop stronger multimodal AI self-efficacy (Berdida et al., 2025).

*H4*: Multimodal AI Literacy has a significant positive effect on Multimodal AI Self-Efficacy.

Zhou et al. (2025), studying engineering undergraduates, found that critical thinking significantly mediated the relationship between GenAI usage competence and AI self-efficacy, indicating that critical thinking and AI self-efficacy are interdependent and jointly affect learning outcomes and creativity. The study suggests that students with stronger critical thinking are more confident in using AI tools, better mastering operational logic and application boundaries. As analytical and evaluative skills improve, students are more likely to perceive themselves as effectively controlling AI tools, thereby enhancing AI self-efficacy. Similar evidence comes from Li et al. (2023), who found in the context of translation technology learning that critical thinking can influence translation technology competence via academic self-efficacy, demonstrating that higher-order thinking strengthens students’ beliefs in their task competence and promotes performance.

*H5*: Critical Thinking has a significant positive effect on Multimodal AI Self-Efficacy.

Regarding resilience and multimodal AI self-efficacy, Zhai and Li (2025), in an AI-enhanced learning context with 505 Chinese-speaking Malaysian learners, found that resilience and self-efficacy are both important predictors of student engagement, with resilience positively predicting self-efficacy. This indicates that in AI learning environments, students with higher resilience are more likely to develop confidence in AI usage.

*H6*: Resilience has a significant positive effect on Multimodal AI Self-Efficacy.

Dang (2026), in a sample of Vietnamese undergraduates, found that among AI literacy, AI attitude, and AI self-efficacy, only AI self-efficacy significantly predicted higher GPA categories, suggesting that students confident in their AI abilities are more likely to achieve better academic performance.

*H7*: Multimodal AI Self-Efficacy has a significant effect on Academic Performance.

### 2.6 Mediating role of cognitive engagement

Cognitive engagement refers to students’ focus, deep processing, and sustained involvement in learning. Studies have shown a significant positive relationship between AI literacy and learning engagement. He et al. (2025), in a sample of 2,029 Chinese nursing students, found that AI literacy was positively correlated with learning engagement (*r* = 0.435, *p* < 0.01) and could directly predict engagement. Therefore, students with higher multimodal AI literacy are more likely to maintain higher levels of cognitive engagement in AI-supported learning environments.

*H8*: Multimodal AI Literacy has a significant effect on Cognitive Engagement.

Regarding critical thinking, prior research demonstrates a significant association with cognitive engagement. Huang et al. (2025) noted that critical thinking enhances students’ active participation and deep involvement in learning processes. Students with stronger critical thinking tend to question, compare evidence, evaluate perspectives, and engage in deep reflection, rather than passively accepting knowledge. This higher-order cognitive processing enhances their sense of meaning and control over learning tasks, promoting sustained engagement.

*H9*: Critical Thinking has a significant effect on Cognitive Engagement.

Regarding resilience, research shows a positive relationship with cognitive engagement. Zhai and Li (2025) found that in AI-assisted Chinese learning contexts, students with higher resilience were more likely to remain engaged in technology-supported learning. Cognitive engagement represents a key component of engagement, reflecting students’ psychological investment in understanding materials, solving problems, and completing learning tasks. Resilient students are more likely to maintain goal-directed behaviors in the face of complex tasks, failure experiences, or learning pressures. Wang et al. (2026) further indicated that psychological resilience not only directly predicts cognitive engagement but may also enhance engagement through academic motivation.

*H10*: Resilience has a significant effect on Cognitive Engagement.

Concerning cognitive engagement and academic performance, Tannoubi et al. (2025), in a study of 488 undergraduates, found a positive correlation between engagement and GPA, with cognitive engagement directly influencing academic success. Students with higher cognitive engagement tend to move beyond surface-level memorization, engaging in analysis, integration, reflection, and deep comprehension, thereby improving knowledge mastery, problem-solving ability, and learning efficiency.

*H11*: Cognitive Engagement has a significant effect on Academic Performance.

He et al. (2025), in a study of nursing undergraduates, found that AI self-efficacy mediated the relationship between AI literacy and learning engagement, suggesting that students who believe in their ability to effectively use AI tools are more likely to invest cognitively in learning. For ECE students, confidence in managing multimodal AI tools promotes greater cognitive investment in lesson planning, activity design, and instructional material analysis.

*H12*: Multimodal AI Self-Efficacy has a significant effect on Cognitive Engagement.

### 2.7 Research model

**Figure 1:**
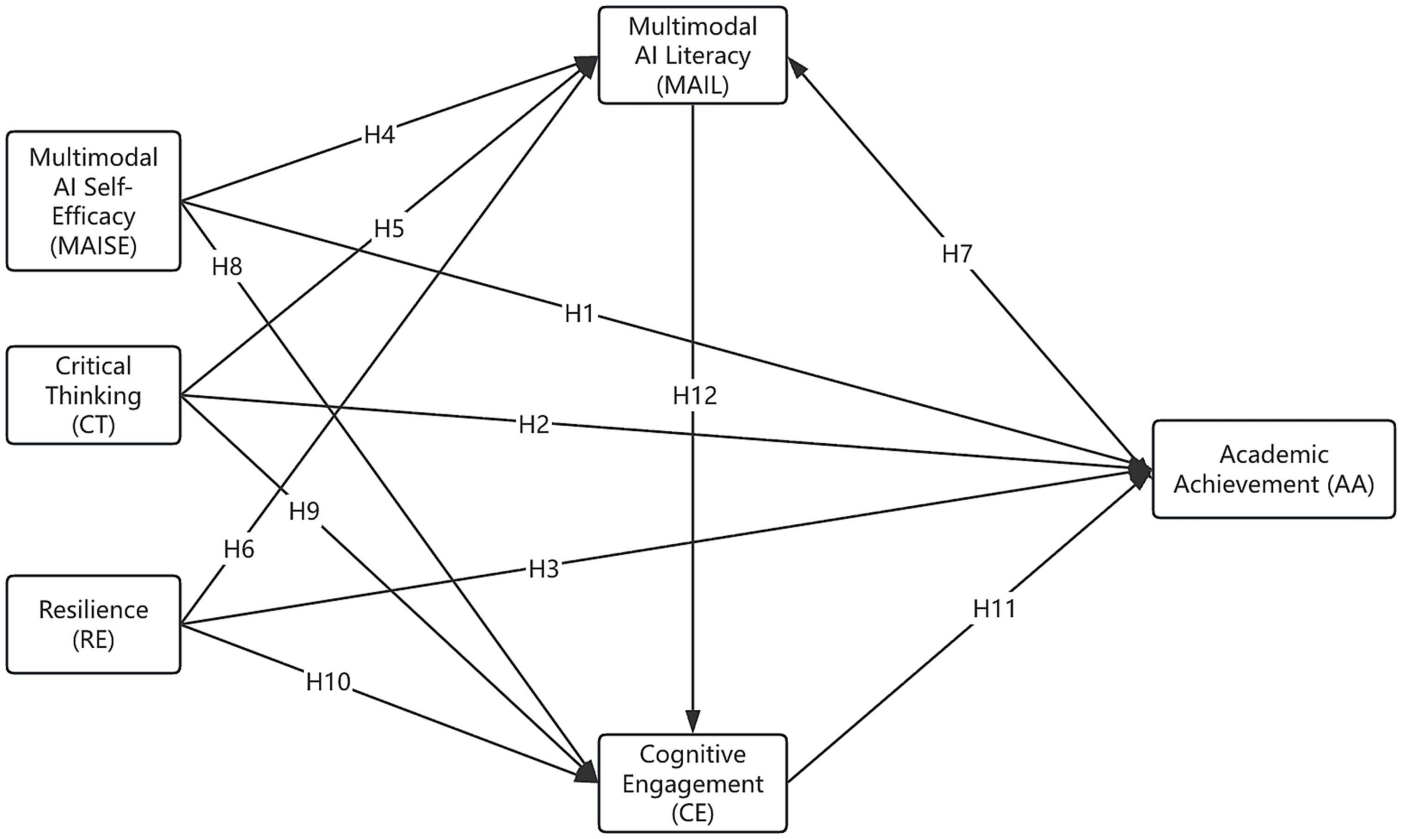
Conceptual model of academic performance for Early Childhood Education students in multimodal AI learning contexts, including three independent variables (Multimodal AI Literacy, Critical Thinking, Resilience), two mediators (Multimodal AI Self-Efficacy, Cognitive Engagement), and one dependent variable (Academic Performance).

As shown in Figure 1, this study constructs a model of academic performance for ECE students in multimodal AI learning contexts, based on three dimensions: technical literacy, cognitive judgment, and psychological adaptation. The model includes three independent variables—multimodal AI literacy, critical thinking, and resilience; two mediating variables—multimodal AI self-efficacy and cognitive engagement; and one dependent variable, academic performance.

## 3 Methodology

### 3.1 Participants and procedure

Participants in this study were undergraduate students majoring in Early Childhood Education (ECE) from universities in Gansu Province, China. This population was selected for several reasons. First, as future early childhood educators, their multimodal AI literacy and cognitive competencies will directly affect the quality of educational practice. Second, compared with engineering or information technology students, ECE students face more prominent interdisciplinary integration challenges when learning AI, which increases the research value. Third, Gansu Province, as a representative region in western China, exhibits a typical level of digital development in education, enhancing the contextual interpretability and practical relevance of the study’s findings. An online questionnaire survey was employed, and participants were recruited via random sampling among ECE students. During data collection, researchers explained the study’s objectives and completion requirements to participants, and questionnaires were completed with informed consent. Participation was voluntary and anonymous, with no personally identifiable information collected, ensuring privacy and data confidentiality. A total of 458 valid responses were collected. Regarding gender distribution, the sample was predominantly female, with approximately 85% female and 15% male students, reflecting a notable gender imbalance favoring females.

### 3.2 Measurement instruments

A structured questionnaire was used as the measurement instrument, divided into two parts. The first part collected participants’ demographic information, including gender, year level, university, and experience with multimodal AI tools. The second part measured the six core constructs of the research model: Multimodal AI Literacy (MAIL), Critical Thinking (CT), Resilience (RE), Multimodal AI Self-Efficacy (MAISE), Cognitive Engagement (CE), and Academic Performance (AP). To ensure theoretical grounding and content validity, this study primarily adopted mature scales widely used or validated in prior domestic and international research, with minor wording adjustments to fit the ECE context and multimodal AI learning environment. All items, except for demographic questions, were scored using a 7-point Likert scale (1 = “strongly disagree” to 7 = “strongly agree”), with higher scores indicating higher levels on the corresponding constructs.

Specifically, Multimodal AI Literacy was measured using the Artificial Intelligence Literacy Scale developed by Wang et al. (2026), which includes 12 items covering four dimensions: awareness, usage, evaluation, and ethics. Critical Thinking was measured using an adapted version of the California Critical Thinking Disposition Inventory (CCTDI) developed by Facione et al. (1994). Resilience was assessed with the Brief Resilience Scale (BRS) developed by Smith et al. (2008), consisting of 6 items measuring individuals’ ability to recover from stress and setbacks. Multimodal AI Self-Efficacy was measured following Hornberger et al.’s AI self-efficacy scale. Cognitive Engagement was measured using an adapted version of the Cognitive Engagement Scale proposed by Miller et al. (1996). Academic Performance was measured via self-reported academic task performance, referencing the Academic Task Performance Scale used in higher education contexts by van Zyl et al. (2024), consisting of 7 items covering time management and task efficiency.

### 3.3 Data analysis method

Data were analyzed using SPSS and SmartPLS. First, SPSS was used to preprocess the sample data, including missing value inspection and outlier detection, ensuring data completeness and clarity of basic characteristics. Reliability of the measurement scales was assessed using Cronbach’s *α* to evaluate internal consistency. Second, Partial Least Squares Structural Equation Modeling (PLS-SEM) was conducted using SmartPLS. The measurement model was first evaluated, including item loadings, composite reliability (CR), and average variance extracted (AVE), to assess convergent validity. Discriminant validity among latent variables was examined using the Heterotrait-Monotrait (HTMT) ratio. After confirming acceptable reliability and validity, the structural model was assessed. Path coefficients (*β*), coefficients of determination (*R*^2^), and effect sizes (*f*^2^) were used to evaluate the strength and explanatory power of relationships between variables. Bootstrapping procedures were applied to test the significance of paths. Additionally, mediating effects were analyzed to examine the multiple chain mediation roles of multimodal AI self-efficacy and cognitive engagement between independent variables and academic performance.

## 4 Data analysis and results

### 4.1 Descriptive analysis

As shown Table 1, all constructs exhibit similar central tendencies, with means ranging from 4.19 (Multimodal AI Literacy) to 4.28 (Academic Performance). Regarding dispersion, standard deviations range from 1.47 (Multimodal AI Literacy) to 1.71 (Resilience), indicating moderate variability around the means. Skewness values are all slightly negative (−0.10 to −0.20), suggesting a slightly left-skewed distribution, but overall approximately symmetric. Kurtosis values are negative (−0.76 to −1.13), indicating platykurtic distributions with relatively few extreme values. Overall, the measurement items for all constructs show reasonable distributions and moderate variability, without substantial skewness or kurtosis issues.

**Table 1:** Descriptive statistics.

Construct	Minimum	Maximum	Mean	Standard deviation	Skewness	Kurtosis
MAIL	1.00	7.00	4.19	1.47	−0.11	−0.80
RE	1.00	7.00	4.24	1.71	−0.13	−1.13
CE	1.00	7.00	4.21	1.50	−0.16	−0.76
CT	1.00	7.00	4.22	1.61	−0.10	−0.94
AA	1.00	7.00	4.28	1.64	−0.19	−0.96
MAISE	1.00	7.00	4.24	1.60	−0.20	−0.93

### 4.2 Measurement model assessment

This study assessed the reliability and convergent validity of the latent constructs using Cronbach’s alpha, composite reliability (ρ_a and ρ_c), and average variance extracted (AVE). As shown in Table 2, all constructs demonstrate good internal consistency. Specifically, Cronbach’s alpha values range from 0.831 to 0.921, well above the recommended threshold of 0.70 (Hair et al., 2010), indicating high internal consistency. Composite reliability values ρ_a and ρ_c range from 0.832 to 0.922 and 0.876 to 0.937, respectively, exceeding the 0.70 threshold (Hair et al., 2010), further confirming construct reliability. In terms of convergent validity, AVE values range from 0.542 to 0.726, all above the 0.50 criterion (Hair et al., 2010), indicating that the latent constructs adequately explain the variance of their indicators and exhibit good convergent validity.

**Table 2:** Construct reliability and validity.

Construct	Cronbach’s alpha	Composite reliability (rho_a)	Composite reliability (rho_c)	Average variance extracted (AVE)
AA	0.921	0.922	0.937	0.68
CE	0.854	0.855	0.891	0.578
CT	0.89	0.894	0.916	0.646
MAIL	0.831	0.832	0.876	0.542
RE	0.906	0.906	0.93	0.726
MAISE	0.889	0.891	0.916	0.644

As shown in Table 3, all HTMT values among constructs are below the stringent threshold of 0.85 (Chin, 1998), indicating good discriminant validity. Pairwise HTMT values range from 0.310 to 0.677, with the highest value between CE and CT (0.677), still well below the threshold, and the lowest between CT and MAIL (0.310), indicating a high degree of differentiation. Other construct combinations (e.g., AA and CE, MAISE and CE) also fall within reasonable ranges, showing no potential discriminant validity issues.

**Table 3:** HTMT (discriminant validity).

Construct	AA	CE	CT	MAIL	RE	MAISE
AA						
CE	0.612					
CT	0.567	0.677				
MAIL	0.474	0.584	0.310			
RE	0.447	0.410	0.340	0.431		
MAISE	0.611	0.663	0.584	0.576	0.534	

### 4.3 Structural model assessment

As shown in Table 4, all predictor variables exhibit VIF values below the common threshold of 3.3, indicating no serious multicollinearity issues (Kock, 2015). Specifically, VIF values across paths range from 1.138 to 2.021, with the highest value for the CE → AA path (2.021) and the lowest for the CT → SE path (1.138), all far below the critical value. This indicates that independent variables are not highly correlated and do not significantly distort path coefficient estimates.

**Table 4:** Collinearity evaluation between the predictor constructs by inner VIF values.

Construct	AA	CE	MAISE
AA			
CE	2.021		
CT	1.694	1.384	1.138
MAIL	1.510	1.370	1.199
RE	1.351	1.348	1.228
MAISE	1.964	1.860	

According to Falk and Miller (1992), an *R*^2^ value of 0.10 is considered the minimum acceptable threshold for endogenous constructs in behavioral research. As shown in Table 5, *R*^2^ values for endogenous constructs are at a moderate level, indicating good explanatory power. Specifically, *R*^2^ for AA is 0.434 (adjusted *R*^2^ = 0.428), suggesting that the antecedent variables explain approximately 43.4% of the variance in academic performance. *R*^2^ for CE is 0.505 (adjusted *R*^2^ = 0.501), indicating strong explanatory power, while *R*^2^ for SE is 0.462 (adjusted *R*^2^ = 0.459), showing moderate-to-high explanatory strength. According to Hair et al. (2019), *R*^2^ values of 0.25, 0.50, and 0.75 represent weak, moderate, and strong explanatory power, respectively. In this study, CE approaches 0.50, demonstrating ideal explanatory capacity, while AA and MAISE also achieve moderate explanatory levels.

**Table 5:** *R*^2^ and *R*^2^ adjusted.

Construct	*R*-square	*R*-square adjusted
AA	0.434	0.428
CE	0.505	0.501
MAISE	0.462	0.459

To further evaluate the impact strength of exogenous variables on endogenous constructs, effect size (*f*^2^) was calculated. As shown in Table 6, effect sizes vary across paths. According to Cohen (1988), *f*^2^ values of 0.02, 0.15, and 0.35 represent small, medium, and large effects, respectively. Most paths show small effect sizes, such as CE → AA (*f*^2^ = 0.030), RE → AA (*f*^2^ = 0.021), and MAISE → AA (*f*^2^ = 0.040). Some paths, e.g., MAIL → AA (*f*^2^ = 0.014) and RE → CE (*f*^2^ = 0.002), fall below 0.02, indicating negligible effects. Notably, CT → CE (*f*^2^ = 0.225) and CT → MAISE (*f*^2^ = 0.216) demonstrate medium effects, suggesting that CT plays a relatively important explanatory role for these endogenous variables. Additionally, MAIL → MAISE (*f*^2^ = 0.143) is near the medium effect threshold, indicating an effect between small and medium.

**Table 6:** The result of f2.

Construct	AA	CE	MAISE
AA			
CE	0.030		
CT	0.054	0.225	0.216
MAIL	0.014	0.102	0.143
RE	0.021	0.002	0.098
MAISE	0.040	0.056	

Stone–Geisser *Q*^2^ values were also evaluated using blindfolding. *Q*^2^ values greater than 0 indicate predictive relevance for endogenous constructs. As shown in Table 7, *Q*^2^ values for AA, CE, and MAISE are 0.291, 0.287, and 0.294, respectively, all significantly above 0, indicating good predictive relevance of the structural model for these endogenous constructs.

**Table 7:** The result of Q2.

Construct	SSO	SSE	*Q*^2^ (=1-SSE/SSO)
AA	3206.000	2271.961	0.291
CE	2748.000	1959.665	0.287
MAISE	2748.000	1940.395	0.294

### 4.4 Hypothesis testing

**Figure 2:**
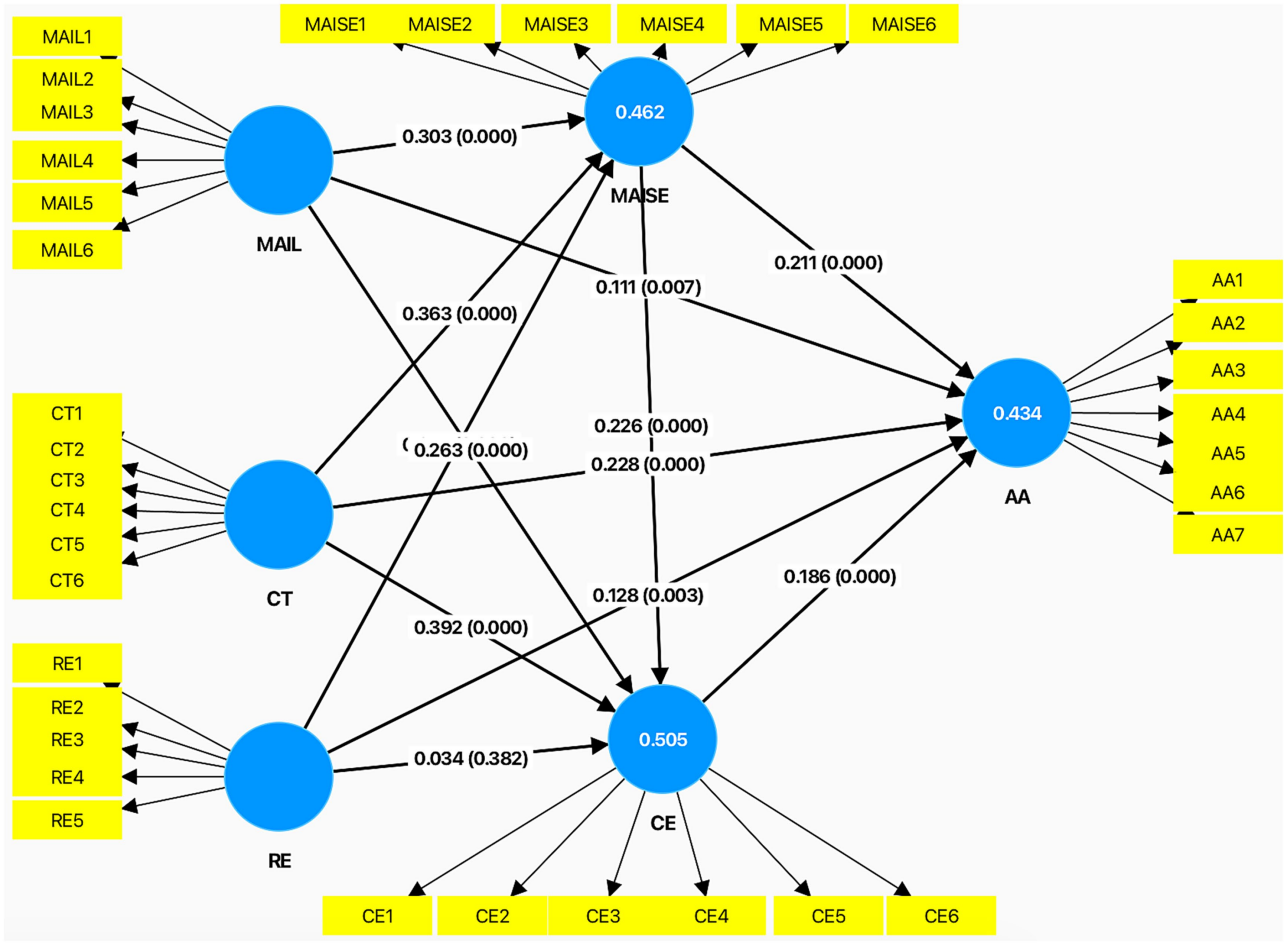
Structural model with path coefficients (*β*), *t*-values, and significance levels (*p*-values) showing the relationships among Multimodal AI Literacy, Critical Thinking, Resilience, Multimodal AI Self-Efficacy, Cognitive Engagement, and Academic Performance.

Partial Least Squares Structural Equation Modeling (PLS-SEM) with bootstrapping procedures was used to test the structural model paths. Table 8 and Figure 2 present the estimated path coefficients (*β*), *t*-values, and significance levels (*p*-values). Results indicate that most hypothesized relationships are empirically supported. Specifically, CE → AA exhibits a significant positive effect (*β* = 0.186, *t* = 3.564, *p* < 0.001), and CT → AA also shows a significant positive effect (*β* = 0.228, *t* = 4.828, *p* < 0.001). Additionally, CT significantly affects CE (*β* = 0.392, *t* = 10.302, *p* < 0.001) and MAISE (*β* = 0.363, *t* = 10.125, *p* < 0.001), indicating the key role of CT in the model. For MAIL-related paths, MAIL positively influences AA (*β* = 0.111, *t* = 2.677, *p* < 0.01), CE (*β* = 0.263, *t* = 6.722, *p* < 0.001), and MAISE (*β* = 0.303, *t* = 8.172, *p* < 0.001), demonstrating its stable effect on multiple endogenous variables.

**Table 8:** Results of hypotheses test.

Construct	Original sample (O)	Sample mean (M)	Standard deviation (STDEV)	*T* statistics (|O/STDEV|)	*p* values
CE >AA	0.186	0.187	0.052	3.564	0.000
CT >AA	0.228	0.227	0.047	4.828	0.000
CT >CE	0.392	0.394	0.038	10.302	0.000
CT >MAISE	0.363	0.363	0.036	10.125	0.000
MAIL >AA	0.111	0.111	0.042	2.677	0.007
MAIL >CE	0.263	0.265	0.039	6.722	0.000
MAIL >MAISE	0.303	0.305	0.037	8.172	0.000
RE >AA	0.128	0.128	0.042	3.018	0.003
RE >CE	0.034	0.034	0.039	0.874	0.382
RE >MAISE	0.255	0.255	0.041	6.265	0.000
SE >AA	0.211	0.211	0.055	3.869	0.000
SE >CE	0.226	0.225	0.045	5.081	0.000

For RE, significant effects were observed on AA (*β* = 0.128, *t* = 3.018, *p* < 0.01) and MAISE (*β* = 0.255, *t* = 6.265, *p* < 0.001), but not on CE (*β* = 0.034, *t* = 0.874, *p* = 0.382), indicating that this path hypothesis was not supported. Furthermore, MAISE significantly affects AA (*β* = 0.211, *t* = 3.869, *p* < 0.001) and CE (*β* = 0.226, *t* = 5.081, *p* < 0.001), further confirming its key role in the model.

## 5 Discussion

### 5.1 Key findings and analysis

The results of this study indicate that multimodal AI literacy, critical thinking, and resilience all have significant positive effects on the academic performance of Early Childhood Education (ECE) students, which aligns with findings from previous research (Du et al., 2024; Wang et al. (2026); Ren et al., 2020; Orhan, 2022; Cai and Meng, 2025). In multimodal AI-supported learning environments, ECE students are required to interact with AI-generated texts, images, stories, videos, and teaching materials. Therefore, students with higher multimodal AI literacy are better able to understand the functions and limitations of AI tools, evaluate the appropriateness of AI-generated materials, and transform these resources into meaningful academic and professional learning outcomes.

Furthermore, resilience, as a key psychological resource, helps students maintain sustained engagement and optimize learning strategies when faced with uncertainties and challenges in AI learning environments, thereby enhancing academic performance. However, in contrast to some studies suggesting that resilience directly promotes learning engagement (Zhai and Li, 2025), the present study finds that its direct effect on cognitive engagement is not significant, suggesting that resilience primarily exerts its influence through indirect pathways in this context. This discrepancy may stem from differences in the sample population or learning environment.

Further analysis indicates that multimodal AI self-efficacy and cognitive engagement function not only as independent mediators but also as a significant chain mediation pathway. This pathway is consistent with Social Cognitive Theory, which emphasizes that personal capabilities and self-beliefs shape learning behaviours and learning outcomes. Multimodal AI self-efficacy represents students’ belief that they can effectively manage AI-supported learning tasks, whereas cognitive engagement reflects their actual mental investment in learning, including analysis, reflection, elaboration, and problem-solving. Therefore, this study suggests that, in AI-supported ECE learning contexts, academic performance depends not only on what students know, but also on whether they believe they can use AI effectively and whether they are deeply engaged in the cognitive processing of learning tasks. This finding is consistent with previous research showing that AI self-efficacy can significantly predict academic performance (Dang and Nguyen, 2025).

### 5.2 Research contributions

This study contributes to theory, practice, and methodology by constructing and validating a chain mediation model centered on multimodal AI self-efficacy and cognitive engagement, systematically revealing how multimodal AI literacy, critical thinking, and resilience jointly influence the academic performance of ECE students.

The study addresses the limitations of prior research that primarily focused on single variables or simple direct paths, proposing a systematic explanatory framework based on Social Cognitive Theory: “Competence (Multimodal AI Literacy & Critical Thinking) → Belief (Multimodal AI Self-Efficacy) → Behavior (Cognitive Engagement) → Outcome (Academic Performance)”. This framework not only confirms the direct effects of multimodal AI literacy and critical thinking on academic performance but also uncovers their multi-path and chain-mediated effects through AI self-efficacy and cognitive engagement, deepening understanding of the mechanisms underpinning academic performance in AI-supported learning contexts. Additionally, this study broadens the scope of AI education research by focusing on ECE students rather than the commonly studied engineering or IT majors, emphasizing the critical role of critical thinking in evaluating and filtering AI-generated content, thereby offering new theoretical insights for non-technical disciplines.

The findings provide actionable implications for curriculum design and instructional practices in higher education. Results indicate that improving students’ AI tool operation skills alone is insufficient to significantly enhance learning outcomes. Developing critical thinking and multimodal AI self-efficacy is crucial to promote deep cognitive engagement. Specifically, universities should shift from “technical training” to “competency development” by designing contextualized, problem-based learning tasks that guide students in analyzing and evaluating AI-generated content to enhance learning quality. Moreover, attention should be paid to learners’ psychological adaptability. Although resilience does not directly impact cognitive engagement, it can indirectly enhance academic performance by strengthening self-efficacy. Thus, instructional practices should provide supportive learning environments and appropriately challenging tasks to improve students’ adaptability and sustained engagement when facing technological uncertainties, facilitating the transition from merely “using multimodal AI” to “effectively leveraging multimodal AI for learning.”

This study employs PLS-SEM to systematically test complex multivariate relationships, suitable for exploratory research and multi-mediator model analysis. By constructing a chain mediation model involving multimodal AI self-efficacy and cognitive engagement, the study reveals a staged mechanism from competence factors to learning outcomes, offering stronger explanatory power than traditional single-mediator or direct-effect models.

### 5.3 Limitations and future research

Despite systematically revealing the mechanism by which multimodal AI literacy, critical thinking, and resilience influence academic performance through AI self-efficacy and cognitive engagement, several limitations warrant attention. First, the cross-sectional design and self-reported questionnaire data, while effective for examining relationships, limit causal inference. Future research could adopt longitudinal or experimental designs to more accurately test causal paths and dynamic processes. Second, the sample was drawn from ECE students in universities in Gansu Province, China. Although regionally representative, the generalizability of findings to other cultural and educational contexts requires further validation. Additionally, the high proportion of female participants, while reflective of the gender distribution in ECE, may influence the broader applicability of results. Future studies could expand sample diversity, including cross-regional or cross-cultural comparisons, to enhance external validity. Third, the measurement of cognitive engagement was relatively simplified, potentially not capturing its multidimensional structure. Future research could employ more comprehensive scales measuring emotional, behavioral, and cognitive engagement for a more nuanced analysis. Finally, this study primarily relies on learners’ subjective perceptions of AI usage, without incorporating actual behavioral data. Future research could combine learning analytics, such as system logs, operational records, or learning trajectory data, to examine how human-AI interactions impact learning behaviors and outcomes.

## 6 Conclusion

This study, focusing on ECE students, constructed and tested the mechanism of academic performance formation in multimodal AI learning contexts based on Social Cognitive Theory. Results indicate that multimodal AI literacy, critical thinking, and resilience all positively influence academic performance, with critical thinking playing a particularly prominent role. Moreover, multimodal AI self-efficacy and cognitive engagement serve as key mediators, not only bridging multiple variables to academic performance individually but also forming a significant chain mediation pathway. Specifically, learners’ multimodal AI literacy and cognitive abilities enhance self-efficacy, which in turn promotes cognitive engagement, ultimately improving academic performance. Resilience, although significantly enhancing academic performance and self-efficacy, does not directly affect cognitive engagement, suggesting that its influence primarily occurs through indirect paths. Overall, this study enriches theoretical explanations of academic performance formation in AI learning contexts and provides new empirical evidence for understanding learning processes among non-technical majors in intelligent learning environments. The findings offer valuable insights for universities to optimize curriculum design and enhance students’ multimodal AI literacy and comprehensive competencies. This study extends Social Cognitive Theory to the context of multimodal AI-supported ECE teacher education, thereby enriching the explanatory scope of the theory. Specifically, multimodal AI literacy, critical thinking, and resilience serve as students’ competencies and psychological resources, which further influence academic performance through multimodal AI self-efficacy and cognitive engagement. This mechanism helps explain how students’ technical competence, cognitive judgment, psychological adaptability, learning beliefs, and learning behaviours jointly shape academic outcomes in AI-enhanced learning environments.

## Data Availability

The raw data supporting the conclusions of this article will be made available by the authors, without undue reservation.
